# A protocol for a scoping review to identify methods used in clinical practice to assess wound odour

**DOI:** 10.12688/hrbopenres.13739.1

**Published:** 2023-09-13

**Authors:** Georgina Gethin, Kimberly LeBlanc, John D Ivory, Caroline McIntosh, Damien Pastor, Enda Naughten, Chloe Hobbs, Barry McGrath, Stephen Cunningham, Lokesh Joshi, Suzanne Moloney, Sebastian Probst

**Affiliations:** 1 School of Nursing and Midwifery, University of Galway, Galway, Ireland; 2 University of Galway, Alliance for Research and Innovation in Wounds, Galway, Ireland; 3 Geneva School of Health Sciences, HES-SO University of Allied Sciences and Arts, Geneva, Switzerland; 4 McGill University, Quebec, Canada; 5 School of Health Sciences, University of Galway, Galway, Ireland; 6 Department of Dermatology and Venerology, University Hospital Geneva, Geneva, Switzerland; 7 School of Biological and Chemical Sciences, University of Galway, Galway, Ireland; 8 HS Ireland, Hidradenitis Suppurativa Association, County Clare, Ireland

**Keywords:** Wound; odour/odor; malodour; assessment; smell

## Abstract

**Objective:** The objective of this scoping review is to map, from wound assessment tools and other literature, the current methods used to assess wound odour in order to answer the following question: Which methods of assessment, validated or otherwise, are currently used in wound assessment tools to assess wound odour?

**Introduction:** Wound assessment includes not only details of the condition of the wound bed but also evaluation of symptoms associated with the wound including that of odour. Odour is cited by clinicians, patients and carers as one of the most distressing wound symptoms. However, there is no consensus on a preferred method to assess odour thus negatively impacting the internal and external validity of many clinical trials and minimising the ability to perform meta-analysis.

**Eligibility criteria:** Any wound assessment tool or framework that includes assessment of wound odour in any wound aetiology and in any care setting. Any systematic or scoping review that includes assessment of wound odour in any wound aetiology and in any care setting. No limits on date of publication or language will be applied.

**Methods:** We will employ the Preferred Reporting Items for Systematic Review and Meta-Analyses extension for scoping reviews (PRISMA-ScR) guidelines for this scoping review and base its structure on the framework proposed by Arksey and O’Malley.

**Results:** A narrative format will summarise extracted data and provide an overview of tools used to assess wound odour. A PRISMA diagram will outline the results of the search strategy. The identified tools will be summarised in table format and stratified according to methods used.

**Conclusion:** The result of this scoping review will be a list of methods used to assess odour in wounds and will be used to inform a subsequent Delphi study to gain consensus on the preferred method to assess wound odour.

## Introduction

Odour is cited by patients and clinicians as one of the most distressing wound symptoms. Such odour is described in vivid and repulsive language and indicates the profound impact it has on the individual ^1– 3^. Terms such as ‘rotting flesh’, ‘nauseating’ ‘putrid’, rotting meat’, ‘foul’ are just some of the descriptors reported in the literature. When individuals have wound odour it can lead to social isolation, fear of social events or family visits, being unable to work, reduced quality of life, feelings of low self-esteem and hopelessness in trying to manage the odour ^1^. Wound odour can occur in various types of wounds including vascular ulcers (arterial or venous leg ulcer), pressure ulcers, diabetic foot ulcers, malignant fungating wounds and or recurrent abscesses and fistulas such as in hidradenitis suppurativa.

Two factors are generally recognised as contributing to malodour in wounds, the first being degradation of devitalised wound tissue and the second being the production of malodourous metabolic end products by both aerobic and anaerobic bacteria ^4^. All wounds are vulnerable to colonization by bacteria from both endogenous sources and from the surrounding environment. The moist, nutrient-rich conditions found in the wound provide the optimal environment for the growth and proliferation of bacteria which have the potential to cause damage to the host tissue as the wound becomes infected ^5^. As bacteria colonise the wound, they produce volatile metabolic end products including short-chain fatty acids (for example; acetic acid which produces a sour odour in the wound), sulphur compounds, putrescine and cadaverine ^6^. This colonisation, once established is typically of a number of bacterial species and in a short period of time can establish as a biofilm ^7^. Biofilm establishment and prevalence is more common in chronic wounds than in acute wounds, with an occurrence of up to 60% within chronic wounds compared to less than 10% of acute wounds ^8^. The exact bacterial and on occasion fungal species present which make up a biofilm may differ from individual to individual with a common core of bacterial species remaining constant owing to their occurrence naturally on the skin. The formation and establishment of a biofilm leads to complications reducing the efficacy of antibiotics and altered localised immune function which both contribute to the complexity of wound management, impacting effective wound healing and increasing wound odour. Of note, odour in wounds may also occur because of using certain types of dressings (i.e. hydrocolloids or skin substitutes).

An international survey among 1,445 clinicians reported odour as being one of the most distressing wound symptoms ^9^. Yet, in the survey only 12% of healthcare professionals assessed odour in routine practice. A systematic review of 105 randomised controlled trials related to venous leg ulceration showed that only eight trials assessed odour and of these 38% (n=3) did not provide details on how this was assessed ^3, 10^. A recent systematic review of topical interventions to manage wound odour reported that of the five studies that met the inclusion criteria, meta-analysis was not possible due in part to different methods of assessment being used and different timing of assessment ^3^.

In the industry field, the perception of, and assessment of odour issues is commonly described by frequency, intensity, duration of exposure, offensiveness, and location (FIDOL) of a smell. Assessing odour can be performed objectively in a standardized, reliable, reproducible way (i.e. using an electronic nose, olfactometer, gas chromatography separation coupled with a mass spectrometer detector analysis) and/or subjectively (Sniffin's Sticks tests). However, in the wound healing field, current tools are subjective, mainly using scales in numeric format which assesses odour intensity while some use descriptive terms also in a scale format such as distance from the wound when odour is detected ^3, 9, 10^. It should be noted that odour assessment is very subjective due to variation in individuals' abilities and sensitivities to detect smell further compounding the challenges in assessing this in practice.

What has remained apparent from the literature is that while odour is the most distressing symptom of a wound, it is poorly researched and understood, but critically there is no consensus on what the preferred method is to assess odour in practice. 

We aim to conduct a Delphi study among clinicians, industry, patients, and carers to gain consensus on the preferred method of odour assessment but first we must synthesise the literature in the field and map the current state of the art as it relates to odour assessment. A preliminary search of MEDLINE, the Cochrane Database of Systematic Reviews and *JBI Evidence Synthesis* was conducted and no current or underway systematic reviews or scoping reviews on the topic were identified.

The overall objective of this scoping review is to map the methods used to assess wound odour as they are presented in wound assessment tools regardless of wound aetiology or stakeholder group to which the tool applies.

## Keywords

Wound; odour/odor; malodour; assessment; smell

## Methods

The proposed scoping review will be conducted in accordance with the Joanna Briggs Institute (JBI) methodology for scoping reviews ^11, 12^ and guided by the Arksey and O’Malley framework with revisions by Levac ^13, 14^. A scoping review is defined as ‘ *a type of evidence synthesis that aims to systematically identify and map the breadth of evidence available on a particular topic, field, concept, or issue, often irrespective of source (ie, primary research, reviews, non-empirical evidence) within or across particular contexts. Scoping reviews can clarify key concepts/definitions in the literature and identify key characteristics or factors related to a concept, including those related to methodological research*’ ^11^. This scoping review protocol was developed through discussion and consensus among the steering group. Although much discussion among the review group has taken place already together with the inclusion of patient representatives in the design of the review and associated data extraction, we will, in line with the recommendations of Arksey and O’Malley continue to review the protocol with any changes fully reported in the final published review.

The framework of Arksey and O’Malley consists of six stages 1) identifying the research question; 2) identifying relevant studies, 3) selecting studies, 4) charting/mapping the data, 5) collating, summarising and reporting results and 6) expert consultation. Each of these stages are presented below.

## Stage 1: Review question

To identify elements under study and create the eligibility criteria, the acronym PCC (Population, Concept, Context) will be used ^11, 12^.

### Population

Any wound assessment tool that includes an evaluation of wound odour by patients, carers and/or clinicians. Any systematic or scoping review that has evaluated wound odour as a primary outcome. We will exclude individual studies that have evaluated wound odour as we propose that these will have been included in previous systematic or scoping reviews. We will check reference lists of included reviews to ensure that valuable sources of odour assessment tools have not been missed.

### Concept

The topic of interest explored is wound-related odour. The characteristics of wound odour may vary based on the type of wound or the type of bacteria or tissue in the wound or the type of treatment being used. We have considered this as introducing significant heterogeneity into the characteristics of odour. However, our focus is on how odour is assessed and although the context may be different the intensity of the odour should still be assessable.

Even in a similar situation, patients, carers and clinicians may rate the intensity of odour different. Our focus is not on how much difference exists but on how this odour was evaluated. We wish to determine if tools have been developed that can be used by all stakeholders or are limited to one group. In determining the data to be extracted in our review we have included in our group two individuals who are also patients both with hidradenitis suppurativa, a chronic disease that is often associated with malodourous discharge and must be managed by the individual.

We have purposely not limited odour assessment to odour intensity. While intensity is the core factor that is assessed in many tools it is possible that some tools assess for example duration or offensiveness. Thus, we will extract the data as presented in the assessment tool and report on such factors in the final review.

### Context

To increase the scope of this scoping review the context explored will be large and will include all care settings without any geographical limitation. We have not placed any limits on clinical or home setting and will determine from the literature if use of any of the assessment tools are limited to these settings. We also have not placed any limits on country as we wish to capture as potentially broad a range of literature as possible.

This has led us to the final primary review question:

*Which methods of assessment, validated or otherwise are currently used in wound assessment tools to assess wound odour?*

Secondary questions to be answered in this review will include:

- What patient (wound) population is the tool designed for?
- Is the tool designed for use by specific individuals e.g. clinicians only?
- Is there evidence of the tool being validated?
- Are the results of the assessment included in an overall wound score?

**Objectives:**

The overall objective of this scoping review is to map the methods used to assess wound odour as they are presented in wound assessment tools regardless of wound aetiology or stakeholder group to which the tool applies.

The secondary objective of this scoping review protocol is to present a transparent process. In particular:

- To systematically search the named databases to identify studies in which the assessment and intensity of odour in any care setting in any country are reported
- To describe information sources of the identified studies reporting odour assessments and their intensity of wounds
- To extract and appraise the data from the included studies about odour assessment and their intensity
- To describe the data extraction process from each of the studies
- To provide a comprehensive summary of the current methods used to assess wound odour as reported in the literature.

## Stage 2: Types of sources

This scoping review will be limited to any study that has reported on the development of a wound assessment tool, whether or not the validity or reliability of that tool has been tested. The tool must be available for use in clinical practice or by patients and must include any form of assessment of wound odour. We will also include any systematic review or scoping review of interventions to manage wound odour in which odour was the primary outcome.

## Search strategy

Relevant keywords and controlled vocabulary will be sought from content experts and located through preliminary searches of Ovid Medline and EBSCO CINAHL. A final search strategy to locate titles and abstracts of articles reporting on methods used to assess wound odour in wounds will then be developed in Ovid Medline, peer reviewed according to the Peer Review of Electronic Search Strategies (PRESS) guidelines ^15^ and adapted for use in the following databases:

- PubMed
- Embase
- EBSCO CINAHL
- Cochrane CENTRAL
- LILACS (Latin American and Caribbean health sciences literature) - Spanish
- LiSSa – health scientific literature

The Bielefeld Academic Search Engine (BASE) will be searched for relevant grey literature.

The search strategy will be piloted to check for appropriateness of keywords and controlled vocabulary terms and edited as necessary to optimise sensitivity and specificity.

Studies published in any language will be included. There are no limitations to date of publication.

## Stage 3: Study selection

Search results will be imported into Rayyan QCRI ^16^ where: titles and abstracts will be screened for inclusion based on eligibility criteria outlined in Table 1. Full text articles of eligible titles and abstracts will then be screened according to the same eligibility criteria ( Table 1) and for reported data relating to assessment of wound odour.

**Table 1.** Eligibility criteria.

Inclusion criteria	Exclusion criteria
**Study types:** Systematic reviews Scoping Reviews Original reports of wound assessment tool development Validation studies of wound assessment tools	Individual intervention studies in which odour was reported as they are likely to have been captured in systematic or scoping reviews.
Studies that report on any tools or strategies, whether validated or otherwise, to assess for odour in wounds across all aetiologies.	Individual studies of any methodology that assess odour as part of the baseline evaluation or as a primary or secondary outcome. We propose that to identify any individual study in which odour was assessed would be far beyond the scope of this review or any other review. But, we propose that individual studies in which odour was assessed should be captured in any systematic or scoping review on the topic area and we will further review all reference lists of identified reviews to ensure we have retrieved all potentially relevant papers.
Any wound assessment tool, framework, guideline/ clinical practice protocol or instrument that includes assessment of odour. A wound of any aetiology assessed in any care setting.	
Time period: No limits on date	
Languages: No limits	

All screening exercises will be carried out independently by two reviewers. Discrepancies will be resolved via discussion between those two reviewers with ongoing disagreement referred to a third reviewer. Both screening rounds will be piloted prior to engaging in the exercise proper.

Following the search, all identified citations will be collated and uploaded into *EndNote version 19 (Clarivate Analytics, PA, USA)* and duplicates removed. Following deduplication all citations will be exported into Rayyan and deduplicated again. Following a pilot test, titles and abstracts will be screened by at least two independent reviewers for assessment against the inclusion criteria. All potentially relevant sources will be retrieved in full. The full text of selected citations will be assessed against the inclusion criteria by at least two independent reviewers. Reasons for exclusion at full text stage will be recorded and reported in the final scoping review. Any disagreements between reviewers at each stage of the process will be resolved through discussion, or in consultation with an additional reviewer/s. The results of entire process will be reported in the final scoping review and presented in a Preferred Reporting Items for Systematic Reviews and Meta-analyses extension for scoping review (PRISMA-ScR) flow diagram ^17^.

## Stage 4: Charting the data

A data extraction form will be developed *a priori* in Microsoft Excel ® and used to capture the following data: see Table 2.

**Table 2.** Data extraction instrument.

Data to be extracted	Descriptor
Citation details	Provide full citation
Type of publication	Systematic review, scoping review etc.
Country of Origin	If available
Name of Tool	Use acronym and full title
Method of odour intensity assessment	E.G. verbal, numeric, image based
Size of scale if used	E.G. 0-10 or 5-point descriptive scale
Direction of scale if used	E.G. was 0 no odour or the least possible odour or was 0 the worst level of the scale
Were other odour descriptors assessed	Please state
If yes to other odour descriptors, please provide details	Details of any other odour characteristics the tool has used
Has the tool been assessed for validity and reliability: please provide details	If a citation to another article which has assessed either validity or reliability or both is provided, then extract the secondary citation details here
In what user group has the validity and reliability been assessed?	E.G., clinicians, patients, carers
In what wound aetiology has the validity and reliability been assessed?	E.G. Venous leg ulcers, malignant fungating wounds
Did the result of odour assessment contribute to an overall wound score or was it a stand-alone assessment	Some wound assessment tools provide a composite score, if so, please provide details
What wound aetiology is the tool designed for?	If the tool is specific to one aetiology, please state or if no aetiology is recommended, please state
Is the tool designed for use in all settings?	
Is the tool designed for use by clinicians only?	
Can the tool be used by patients and or carers?	

Data will be extracted from papers by two or more independent reviewers using a data extraction tool developed by the reviewers. The data extracted will include specific details about the aetiology, study type, tools used and key findings relevant to the review question/s (see Table 2). We will pilot test the data extraction tool against five full text publications among two review authors working independently. Any disagreements will be resolved through discussion and any changes that are necessary to the data extraction form will be discussed with the review group.

A draft extraction form is provided (see Table 2). The draft data extraction tool will be revised as necessary during the data extraction process. Revisions will be reported in the scoping review. Any disagreements between the reviewers will be resolved through discussion, or where necessary with an additional reviewer/s. If necessary, authors of papers will be contacted to request missing or additional data.

## Stage 5: Collating, summarising and reporting the results

The data will be presented diagrammatically, graphically or in tabular form as appropriate. A narrative summary will describe how the results address the reviews objective and question/s.

## Stage 6: Expert consensus

This is an optional stage but as the final objective is to gain consensus on the preferred method to assess wound odour it is important that it is embedded throughout the review process. With this in mind members of the steering group will discuss the review within their networks and during major wound care conference and events throughout 2023. Members of the steering group are currently leading members of various wound care organisations such as European Wound Management Association, Alliance for Research and Innovation in Wounds, Canadian Nurses Association International Skin Tears Advisory Panel, European Pressure Ulcer Panel.

## Discussion

It is important that we move towards systematic and consistent use of measurement tools in the field of wound care. Currently, the lack of core outcome sets and well validated and reliable assessment tools is hampering meta-analysis and confidence in the levels of evidence in the field. This review will contribute in some way towards improving the situation as it applies to wound odour. The problem of wound odour is a significant and debilitating one for patients and carers and it is critical therefore that we try to improve this situation. The findings of this scoping review will inform a Delphi study to gain consensus on the preferred methods to assess wound odour and in so doing will also raise awareness of the need for assessment and the development of interventions to address this problem.

Search strategy dated: 22 ^nd^ March 2023

1. Smell/

2. odo?r*.tw.

3. 1 or 2

4. Odorants/pc [Prevention & Control]

5. (assess* adj3 tool*).tw.

6. (assess* adj3 Framework*).tw.

7. (assess* adj3 protocol*).tw.

8. (assess* adj3 "best practice*").tw.

9. (assess* adj3 guide*).tw.

10. (measur* adj3 Framework*).tw.

11. (measur* adj3 tool*).tw.

12. (measur* adj3 protocol*).tw.

13. (Measur* adj3 guide*).tw.

14. (measur* adj3 "best practice*").tw.

15. 4 or 5 or 6 or 7 or 8 or 9 or 10 or 11 or 12 or 13 or 14

16. 3 and 15

## Data Availability

No data are associated with this article.

## References

[1] Probst S Arber A Faithfull S : Malignant fungating wounds: the meaning of living in an unbounded body. *Eur J Oncol Nurs.* 2013;17(1):38–45. 10.1016/j.ejon.2012.02.001 22459257

[2] Probst S Arber A Trojan A : Caring for a loved one with a malignant fungating wound. *Support Care Cancer.* 2012;20(12):3065–3070. 10.1007/s00520-012-1430-y 22391594

[3] Gethin G Vellinga A McIntosh C : Systematic review of topical interventions for the management of odour in patients with chronic or malignant fungating wounds. *J Tissue Viability.* 2023;32(1):151–157. 10.1016/j.jtv.2022.10.007 36376189

[4] Hayashida K Yamakawa S : Topical odour management in burn patients. *Burns Trauma.* 2021;9:tkab025. 10.1093/burnst/tkab025 34458382 PMC8389170

[5] Bowler PG Duerden BI Armstrong DG : Wound microbiology and associated approaches to wound management. *Clin Microbiol Rev.* 2001;14(2):244–269. 10.1128/CMR.14.2.244-269.2001 11292638 PMC88973

[6] Bowler PG Davies BJ Jones SA : Microbial involvement in chronic wound malodour. *J Wound Care.* 1999;8(5):216–218. 10.12968/jowc.1999.8.5.25875 10531934

[7] Clinton A Carter T : Chronic Wound Biofilms: Pathogenesis and Potential Therapies. *Lab Med.* 2015;46(4):277–84. 10.1309/LMBNSWKUI4JPN7SO 26489671

[8] James GA Swogger E Wolcott R : Biofilms in chronic wounds. *Wound Repair Regen.* 2008;16(1):37–44. 10.1111/j.1524-475X.2007.00321.x 18086294

[9] Gethin G Grocott P Probst S : Current practice in the management of wound odour: An international survey. *Int J Nurs Stud.* 2014;51(6):865–874. 10.1016/j.ijnurstu.2013.10.013 24238490

[10] Gethin G Ivory JD Connell L : External validity of randomized controlled trials of interventions in venous leg ulceration: A systematic review. *Wound Repair Regen.* 2019;27(6):702–710. 10.1111/wrr.12756 31376298

[11] Munn Z Pollock D Khalil H : What are scoping reviews? Providing a formal definition of scoping reviews as a type of evidence synthesis. *JBI Evid Synth.* 2022;20(4):950–952. 10.11124/JBIES-21-00483 35249995

[12] Peters M Godfrey CM Mcinerney P : Scoping Reviews.In: *JBI Manual for Evidene Synthesis*. E. Aromataris and Z. Munn, Editors, Johanna Briggs Institute,2020. 10.46658/JBIMES-20-12

[13] Arksey H O’Malley L : Scoping studies: towards a methodological framework. *Int J Soc Res Methodol.* 2005;8(1):19–32. 10.1080/1364557032000119616

[14] Levac D Colquhoun H O’Brien KK : Scoping studies: advancing the methodology. *Implement Sci.* 2010;5:69. 10.1186/1748-5908-5-69 20854677 PMC2954944

[15] McGowan J Sampson M Salzwedel DM : PRESS Peer Review of Electronic Search Strategies: 2015 Guideline Statement. *J Clin Epidemiol.* 2016;75:40–46. 10.1016/j.jclinepi.2016.01.021 27005575

[16] Ouzzani M Hammady H Fedorowicz Z : Rayyan-a web and mobile app for systematic reviews. *Syst Rev.* 2016;5(1):210. 10.1186/s13643-016-0384-4 27919275 PMC5139140

[17] Tricco AC Lillie E Zarin W : PRISMA Extension for Scoping Reviews (PRISMA-ScR): Checklist and Explanation. *Ann Intern Med.* 2018;169(7):467–473. 10.7326/M18-0850 30178033

