# Supplementary material for: A protocol for a scoping review to identify methods used in clinical practice to assess wound odour
Source: HRB Open Res. 2023 Sep 13;6:54. [Version 1] doi: 10.12688/hrbopenres.13739.1 (PMC11406125; doi:10.12688/hrbopenres.13739.1)
Supplement: Peer review history

# Reviewer report: Reviewer response for version 1

**DOI:** 10.21956/hrbopenres.15029.r37303

**Published:** 2024-09-16

**Authors:** Charles Tilley

**Affiliations:** 1 NYU Rory Meyers College of Nursing, New York, NY, USA

Excellent and timely topic, standardization of wound odor assessment is important for both research and clinical applications.

 In the methodology section, may I suggest you more robustly describe which symptom characteristics of odor assessment you will be reviewing? The Theory of Unpleasant Symptoms describes 4 symptom characteristics: timing, intensity, distress, and quality. Perhaps expanding your last paragraph under "Stage I: Review Question" to include these assessment parameters may help the reader understand which assessment factors you will include.

Is the study design appropriate for the research question?

Yes

Is the rationale for, and objectives of, the study clearly described?

Yes

Are sufficient details of the methods provided to allow replication by others?

Partly

Are the datasets clearly presented in a useable and accessible format?

Yes

Reviewer Expertise:

Palliative wound care; symptom science; palliative oncology; simulation outcomes

I confirm that I have read this submission and believe that I have an appropriate level of expertise to confirm that it is of an acceptable scientific standard, however I have significant reservations, as outlined above.

# Reviewer report: Reviewer response for version 1

**DOI:** 10.21956/hrbopenres.15029.r37300

**Published:** 2024-05-28

**Authors:** Kathleen Finlayson

**Affiliations:** 1 Queensland University of Technology, Brisbane, Queensland, Australia

The article describes a proposed protocol for a scoping review of methods used to assess wound odour,  a symptom known to cause extreme distress to those suffering from chronic, infected and/or malignant wounds. It is thus of importance to review validated methods to assess odour.  

 Some suggestions:  

   in methods,  you note you include any wound assessment tool that includes an evaluation of wound odour - will you be including both quantitative and qualitative evaluation within any assessment method?  or only quantitative tools?  I'd specify.

 In the inclusion criteria:    I'd suggest including individual studies published after the dates of the end of searches within the reviews that you locate - there's likely to be a year or so lapse?  

 In the search strategy:  consider including the term 'symptom' in your searches, as some scales include odour as part of overall symptom measurement,  e.g. Toronto Symptom Assessment Scale

 minor point:    the quoted definition of a scoping review requires a page number with the citation.

Is the study design appropriate for the research question?

Yes

Is the rationale for, and objectives of, the study clearly described?

Yes

Are sufficient details of the methods provided to allow replication by others?

Yes

Are the datasets clearly presented in a useable and accessible format?

Not applicable

Reviewer Expertise:

chronic wounds, symptoms associated with chronic wounds, implementation science

I confirm that I have read this submission and believe that I have an appropriate level of expertise to confirm that it is of an acceptable scientific standard, however I have significant reservations, as outlined above.

# Response

**Published:** 2024-05-28

**Authors:** Georgina Gethin

**Affiliations:** National University of Ireland, Galway, Ireland

Thank you for your review of our protocol which is very helpful.

 The tools will include both quantitative and qualitative methods and the search strategy is open to that. We will make note of it in the final review methods section. 

 If we include the word 'symptom' in the search, from a preliminary search it yields a very large amount of articles with very few being relevant to the review and of these we have captured them using the current strategy. However, we will monitor this as we proceed.

 We will also search for any studies that have been published after the last of the systematic/scoping reviews

 Thanking you

# Reviewer report: Reviewer response for version 1

**DOI:** 10.21956/hrbopenres.15029.r36151

**Published:** 2023-09-29

**Authors:** Victoria Clemett

**Affiliations:** 1 Florence Nightingale Faculty of Nursing and Midwifery, King's College London, London, England, UK

This is a useful review to bring together the wider body of literature and resources on odour assessment for wounds. 

 The population of interest considers wound related odour assessment tools completed patients, carers (including family) and/or clinicians which is approximate for this type of assessment due to the subjectivity of odour assessment and the variation in odour perception by different people. 

 There has been a clear consideration of patient involvement and wound care organisations when undertaking this scoping review. 

 There is a clear plan outlined in how the findings of this scoping review will be used. 

 The methods section in the abstract could be clearer. The six stage process of scoping reviews proposed by Arksey & O’Malley has been identified but it would be useful to also identify your sources for the scoping review within your abstract. 

 I wonder whether it might be beneficial for your scoping review to also screen international wound-care organisations position statements or clinical advice on their websites to see what tools they are recommending for assessing odour.

Is the study design appropriate for the research question?

Yes

Is the rationale for, and objectives of, the study clearly described?

Yes

Are sufficient details of the methods provided to allow replication by others?

Yes

Are the datasets clearly presented in a useable and accessible format?

Not applicable

Reviewer Expertise:

Co creating solutions to accurately assess patients and meet the needs of people living with a wound. This has included undertaking reviews. I have some awareness of odour assessment and perception related to food and nutrition rather than assessing wound odour

I confirm that I have read this submission and believe that I have an appropriate level of expertise to confirm that it is of an acceptable scientific standard.

# Response

**Published:** 2024-05-28

**Authors:** Georgina Gethin

**Affiliations:** National University of Ireland, Galway, Ireland

Thank you for your review of our protocol which is very helpful.

 We will take into account your suggestions re additional sources and reflect this in the final review and will update the abstract.
